# A Net Shape Profile Extraction Approach for Exploring the Forming Appearance of Inclined Thick-Walled Structures by Wire Arc Additive Manufacturing

**DOI:** 10.3390/mi15101262

**Published:** 2024-10-16

**Authors:** Yexing Zheng, Yongzhe Li, Yijun Zhou, Xiaoyu Wang, Guangjun Zhang

**Affiliations:** 1 School of Mechanical Engineering, Southeast University, Nanjing 211189, China; 2 State Key Laboratory of Advanced Welding and Joining, Harbin Institute of Technology, Harbin 150006, China

**Keywords:** wire arc additive manufacturing, structured-laser sensor, inclined thick-walled structure, forming mechanism, image fusion

## Abstract

Wire arc additive manufacturing (WAAM) offers a viable solution for fabricating large-scale metallic parts, which contain various forms of inclined thick-walled structure. Due to the variety of heat dissipation conditions at different positions, the inclined thick-walled structure is a major challenge in fabrication that may produce collapses and defects. However, there is a lack of effective sensing method for acquiring the forming appearance of individual beads in the structure. This paper proposes a novel approach for extracting individual bead profiles during the WAAM process. The approach utilizes a structured-laser sensor to capture the morphology of the surface before and after deposition, thereby enabling an accurate acquisition of the bead profile by integrating the laser stripes. Utilizing the proposed approach, the research investigated the forming mechanism of beads in inclined thick-walled components that were fabricated by various deposition parameters. The width of the overlapping area at the overhanging feature decreased as the layer number increased, while the height of the same area increased. The height of the overlapping area in each layer increased with an increase in deposition current and decreased when the deposition speed was increased. These phenomena suggest that the heat input is a major factor that influences the formation of the overhanging feature. Both the deposition current and deposition velocity influence heat input, and thereby have an effect in enhancing the geometrical accuracy of an overhanging feature. The experimental results indicate that the proposed approach facilitates morphology change investigation, providing a sufficient reference for optimizing deposition parameters.

## 1. Introduction

### 1.1. Background and Motivation

Over the past decades, metal additive manufacturing (MAM) has attracted increasing research attention due to its significant advantages in forming complex metallic structures that are difficult to produce by traditional processes [1]. As a typical technology of MAM, wire arc additive manufacturing (WAAM) employs an electric arc as the heating source, which provides significant heat input for melting the fed metal wire. For this reason, WAAM is suitable for fabricating large-scale components with a medium complexity in geometry at a relatively low cost [2]. According to the number of beads contained in a layer, the basic structure of WAAM can be either a thin-walled structure, i.e., multi-layer single-bead (MLSB) structure or a thick-walled structure, i.e., multi-layer multi-bead (MLMB) structure [3]. Due to the proliferation of WAAM in industry applications, the inclined thick-walled structure more commonly appears in large-scale parts, and its forming quality is critical to the final product.

During the deposition process of a thick-walled component, the variety of structures, the different selection of processing conditions, and the interaction of overlapping beads significantly impact the shape formation process [4,5,6]. The solidification of an overlapping bead undergoes an asymmetric thermal dissipation condition, which differs from that of a flat substrate. Consequently, geometrical deviations from the designed values are produced and may accumulate during fabrication. In particular, the bead deposited at an overhanging edge tends to collapse due to the insufficiency of the supportive structure. The center of an overlapping bead may deviate from the center of the fed wire, which is caused by the asymmetric surface tension of the melt pool [7]. The above-presented facts necessitate a better understanding of the forming mechanism of inclined thick-walled components, which provides a basis for optimizing the process parameters of WAAM [8].

Investigation of the WAAM forming mechanism requires a dedicated sensing enabler that can obtain the geometrical dimension of the deposit. The commonly used visual sensing approaches fall into two categories: active visual sensing approaches, e.g., structured-laser sensing [9], and passive visual sensing approaches, e.g., melt pool monitoring [10,11]. The state-of-the-art research either depicts the surface of the deposit using sensing solutions or observes the geometrical morphology of a component based on metallography. However, the surface of the deposit can be easily affected due to the existence of the neighboring bead in a thick-walled structure. Metallography cannot represent the net shape profile of individual beads. Accordingly, there is a lack of a sensing solution to aggregate the net shape profile of individual beads, which stimulated the research of this paper [12,13,14].

### 1.2. Literature Review

WAAM has been extensively used to fabricate large-scale parts with various deposition patterns [15] for several applications, such as aerospace [16], shipbuilding [17], and defense [18]. The manufacturing process for large-scale parts typically requires significant time. The cooling process between layers, which is seriously time-consuming, is necessary for the process, limiting the deposition speed; otherwise, the large heat input will cause defects [19]. For this reason, stability of deposition is crucial to the part quality. Any geometrical deviation may invalidate the preplanned deposition parameters, which may lead to the failure of fabrication [20]. Due to the complex relationships between various deposition parameters, the inclined thick-walled structure is a major challenge in fabrication that may produce collapses and defects [21]. The deposition parameters should be adjusted when certain geometrical deviations are observed, so that collapses and defects could be reduced.

Process planning and optimization are necessary to both the efficiency and quality of deposition. Modeling the geometries of the deposit is the precondition for process planning and optimization. Various geometric models for single beads have been proposed, including parabolic models [22], circular models [14], trigonometric models [23], and semi-ellipse models [24]. Except for the profile curve fitting, investigating the relationships between deposition parameters and bead morphology characteristics such as bead height and bead width is of vital importance in process planning and optimization. Wang et al. [25] developed an artificial neural network model considering the varying interlayer temperature, wire feed speed and deposition speed to predict the bead profile. Karmuhilan and Sood [26] designed a reverse model using an artificial neural network for selecting deposition parameters based on user-specified bead geometry. When a layer contains multiple beads, the overlapping distance between neighboring beads should be well arranged to enhance the overlapping quality of beads. To this end, a tangent overlapping model was proposed that incorporates the concept of a critical valley between neighboring beads; thus, the central distance of neighboring beads was precisely predicted [27]. Based on the beads overlapping model, Wang et al. [28] proposed a sequential path-planning method using the water-pouring rule for WAAM. Ding et al. [29] developed a path-planning strategy based on the medial axis transformation and detailed its effectiveness for different structures.

Although sufficient attention has been paid during the research to the planning of the process, work related to inclined thick-walled structure is rare. The exploration of the mutual influences between neighboring beads and the forming behaviors of overhanging structure drew little attention in the reported studies [7,30]. For this reason, it is necessary to investigate the forming mechanism of inclined thick-walled structures for optimizing deposition parameters. Investigating the forming mechanism of inclined thick-walled structures necessitates an effective sensing approach. However, traditional measurement methods are unsuitable for acquiring bead geometries due to the intricate conditions [31]. For instance, classical active sensing methods, e.g., structured-laser sensors, can capture the three-dimensional profile of bead surfaces [9]. However, significant errors are usually reported in the case of an MLMB structure since feature points can hardly be identified from a single laser stripe. Passive visual sensing methods employ arc light to illuminate the melt pool without the need for additional auxiliary light sources [10,11]. While passive sensing methods offer low latency, they are rarely used for measuring MLMB structures because the remelting zone of the neighboring bead may influence the edge extraction of the melt pool, which produces errors in the measurement of the deposit geometry.

It can be seen from the literature that the forming mechanism of the inclined thick-walled structure is exceedingly complex, which necessitates a sufficient understanding of how the geometries of beads change with varying deposition parameters. However, classical sensing methods can hardly be applied to measure the geometries of individual beads.

### 1.3. Objective and Content

This paper presents an algorithmic approach for extracting the net shape profile of deposits in a thick-walled component. The novelty of the proposed approach comes from aggregating the structured-laser stripes before and after deposition. Utilizing the novel approach, the forming mechanism of inclined thick-walled components is presented. Based on the net shape profile of individual beads, the forming mechanism of complex structures can be investigated without the need for metallography. The content of the paper is arranged as follows: Section 2 introduces the algorithmic approach. Section 3 presents the details of the implementation and the results of the experimentation. Section 4 discusses the validation results, and Section 5 gives the conclusions.

## 2. The Net Shape Profile Extraction Strategy

### 2.1. Problem Definition

**Figure 1:**
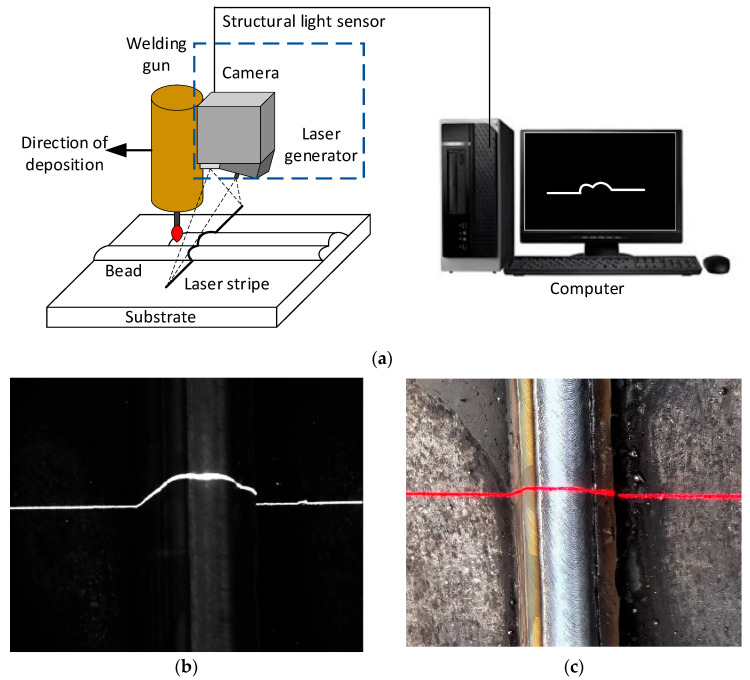
Measuring the geometric information of deposit in an MLMB structure: (**a**) the mechanism of structured-laser sensing; (**b**) a typical laser stripe image; and (**c**) the formed component.

The structured-laser sensor is an effective tool for acquiring three-dimensional information based on the principle of triangulation. For robotic WAAM, a structured-laser sensor using a linear stripe is normally applied for measuring the geometrical dimensions of deposited components. The mechanism of sensing is shown in Figure 1a. The camera aggregates the laser stripe images of a bead, which represents the cross-sectional profile of the deposit surface. The geometries of a bead can be obtained after the feature points are defined and identified from the laser stripe images. For this reason, this sensing method can be used to measure the bead geometries in thin-walled structures, whereas its applicability in the case of thick-walled structures remains uncertain.

A typical laser stripe image of a thick-walled component and the actual situation is shown in Figure 1b and Figure 1c, respectively. It is difficult to accurately identify the intersection point between two beads from the stripe image since the surface morphology of a bead is fused with its neighboring beads, and the toe point can hardly be defined. The situation becomes more complex when the thick-walled component is inclined since every bead is deposited on the interaction of two underpinning beads. Significant errors may be generated if classical image processing approaches are used for measuring bead geometries.

### 2.2. The Developed Approach

**Figure 2:**
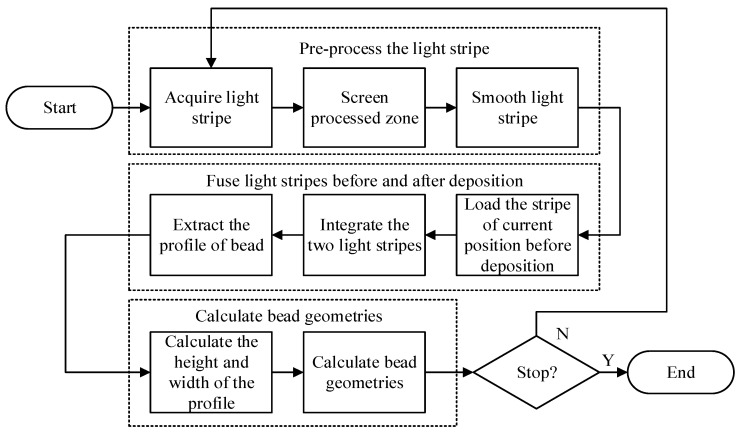
The workflow of the algorithm developed for detecting geometries of beads.

To address the issues mentioned earlier, this work presents a bead profile extraction approach for inclined MLMB deposition. This approach fuses linear-structured laser stripes captured at the same position before and after deposition to obtain the net-forming profile. Subsequently, the differences in profile geometries can be extracted and calculated. Based on the approach, an algorithm was developed to determine the height and width of the bead, as illustrated in Figure 2. The algorithm consists of three stages, including (i) preprocessing of linear-structured laser point cloud data, (ii) fusion of linear-structured laser stripes before and after deposition, and (iii) calculation of the bead geometries. The algorithm processes the linear-structured laser point cloud data of each bead cross-section continuously until all the data are processed.

**Figure 3:**
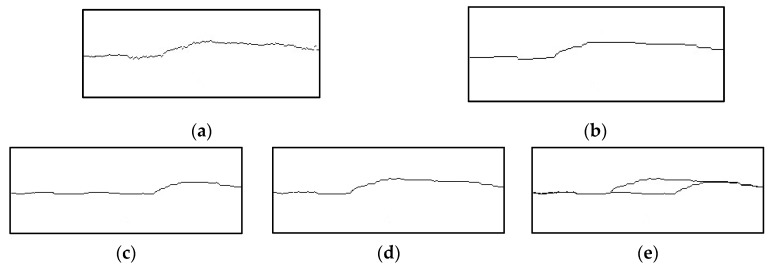
Preprocessing and integration of laser stripes before and after deposition: (**a**) before processing; (**b**) after processing; (**c**) before deposition; (**d**) after deposition, and (**e**) fused laser stripes.

The acquired linear-structured laser stripe should be preprocessed to remove noise and enhance the continuity of the light stripe. To increase the efficiency of processing, the algorithm retains an 18 mm window from the acquired images. A five-point triple smoothing template is then applied to perform sliding average filtering over the light stripe, which helps to eliminate isolated and erroneous points from the laser stripes. As shown in Figure 3a,b, the preprocessed laser stripe data exhibit improved smoothing and denoising effects. This preprocessing method accurately reflects the actual surface topography of the parts without distorting the data, providing a solid foundation for subsequent feature extraction and analysis.

To address the challenge of extracting the intersection points of linear-structured laser stripes in inclined MLMB structures, an approach that fuses linear-structured laser stripes before and after deposition at the same position was developed. This approach allows for the direct acquisition of the current bead profile information. Before deposition, a constant speed is used to scan the original clean area, and the preprocessed linear-structured laser stripe data are sequentially stored at specified time intervals. During deposition, the linear-structured laser stripes of the deposited bead are processed again using the same scanning speed and sampling frequency. Concurrently, the previously stored stripe information from the same spatial position is read from the computer memory and overlaid on the currently processed stripe image, facilitating the image fusion, as shown in Figure 3c–e.

### 2.3. Characteristics of Bead Profile

During the deposition process, the profile of the current bead is influenced by both the adjacent bead and the underlying layer. This interaction complicates the description of the morphology characteristics of the bead profile. In each layer, every bead is deposited on the base of the neighboring bead, except for the first one. If the adjacent bead is wider or higher, the current bead will be padded and become higher. If the neighboring bead is narrower or lower, the current bead will be lower. Consequently, the coupling extent of neighboring beads is considered to accurately describe the morphology characteristics of the beads.

**Figure 4:**
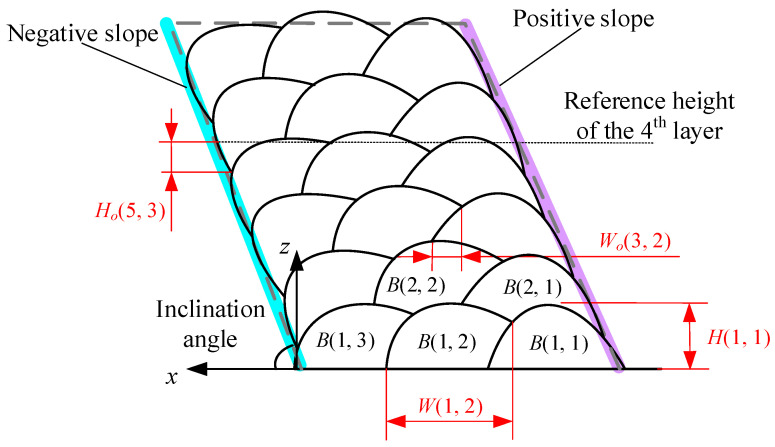
Standard structure and morphology characteristics of the structure.

Based on the fused bead stripes, the algorithm extracts and calculates the height and width differences of the bead profile as well as the height and width of the overlapped areas to calculate the overlap ratios. Specifically, the algorithm applies interpolation to fit curves to the stripe points and calculates the height difference between the upper and lower stripes at specified intervals, selecting the maximum height difference as the bead height. To determine the width, the algorithm identifies the intersection points of the upper and lower stripes and calculates the width difference. The algorithm identifies the intersection point between the stripes of the current and previous stripes, as well as the edge point of the previous bead beneath the current bead profile, to calculate the width difference, which represents the overlapped width. The height difference between the lowest point of the current bead profile and the reference height of the previous layer is used to determine the overlapped height. The overlap ratio is then calculated by dividing the bead height or width by the height or width of the overlapped area. An inclined MLMB structure consisting of six layers with three beads per layer was considered as the target for exploring the morphology characteristics of MLMB beads, as illustrated in Figure 4.

In Figure 4, *B*(*n*,*m*) is the *m*th bead in the *n*th layer. *H*(*n*,*m*) and *W*(*n*,*m*) are the height and width of *B*(*n*,*m*), respectively, while *H_o_*(*n*,*m*) and *W_o_*(*n*,*m*) are the overlapped height and width of *B*(*n*,*m*), respectively. The negative slope is defined as the slope at an acute angle to the substrate, and the opposite slope is the positive slope. The inclination angle is defined as the acute angle between the negative slope and the substrate. The height overlap ratio is defined as follows:

*ε_h_* = *H_o_*(*n*,*m*)/*H*(*n*,*m*) (1)

In particular, if the overlapped height is larger than the bead height (due to a severe collapse), the value of 1 is used as the height overlap ratio. The width overlap ratio is defined as follows:

*ε_h_* = *W_o_*(*n*,*m*)/*W*(*n*,*m*) (2)

## 3. Validation of the Proposed Approach

### 3.1. Experimental Setup

**Figure 5:**
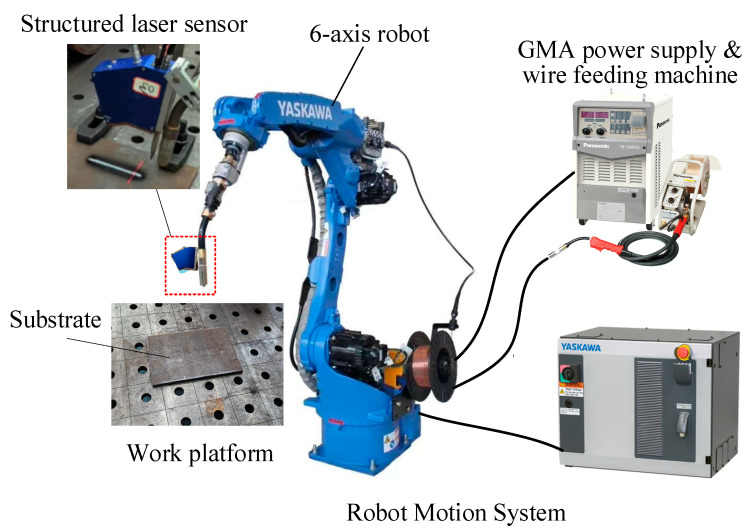
The implemented robotic wire arc additive manufacturing (WAAM) system.

The proposed approach was validated through actual experiments with various parameters. As shown in Figure 5, a robotic WAAM platform was developed for the experimentations. A Motorman AR1730 six-axis robot manufactured by YASKAWA in Shanghai, China, was used as the motion platform, and a Panasonic YD-500FR welding machine equipped with a YW-50KM wire feeding machine, which were both manufactured by Panasonic in Tangshan, China, was used as the deposition power source. The structured-laser sensor was the META SLS-050 V1, which has a built-in image processing unit that allows the user to directly acquire the 2D point cloud data of the laser stripe at a designated position. The accuracy of the measurement system was 0.05 mm. The sensor was placed in the rear of the welding gun. The sensor transmitted the obtained point cloud data to the computer for the subsequent processing steps via Ethernet wireless communication. A computer program based on MATLAB R2022a was designed for image processing. The camera acquires planar checkerboard images and conducts calibration by using the MATLAB Camera Calibration Toolbox. The camera took one photo per second and the stripes were transformed to three-dimensional point cloud data by the program.

Based on the WAAM hardware system platform, several experiments for manufacturing inclined MLMB components were carried out. The experimental materials and process specifications are shown in Table 1. The forming mechanism of inclined MLMB structures was investigated based on the sensing approach. During the planning process, varying deposition parameters, such as deposition voltage, deposition current, deposition speed, and inclination angle, resulted in different bead sizes. Consequently, the distance between each layer and bead varied. Detailed parameters for these experimental settings are provided in the corresponding section.

### 3.2. The Mechanism at Different Positions

A standard structural component was processed using the proposed measure approach to capture the profile of individual deposited beads. The specific deposition parameters are detailed in Table 2. The distance between adjacent beads was calculated by multiplying the width of a single bead by 0.738 [27]. The layer offset distance was calculated by dividing the single bead height by the tangent of the inclination angle.

**Figure 6:**
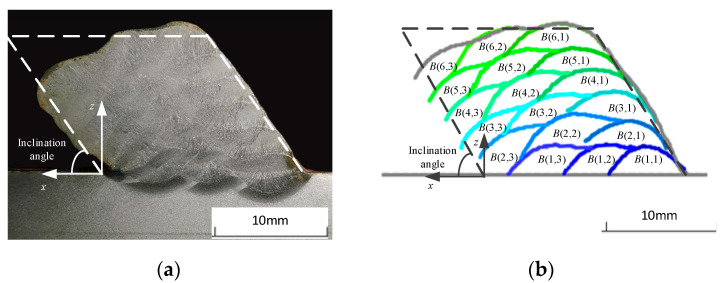
The formed inclined part and the extraction results: (**a**) formed part, and (**b**) extraction results.

**Figure 7:**
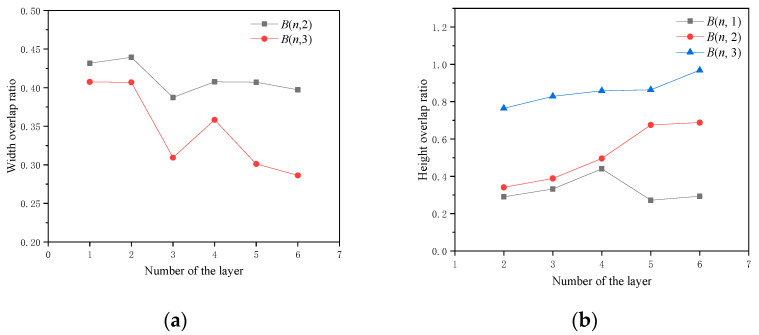
Width (**a**) and height (**b**) overlap ratios of individual beads.

The cross-section of the formed part and the extraction results are shown in Figure 6. The width and height overlap ratios of the beads in the fabricated part are illustrated in Figure 7. Since *B*(*n*,1) did not overlap with any bead, its width overlap ratios were zero. Similarly, the height overlap ratios for *B*(1,*m*) were zero. As n increased, the width overlap ratios of *B*(*n*,2) remained stable at approximately 0.4, while the width overlap ratios of *B*(*n*,3) showed a decreasing trend. The height overlap ratios of *B*(*n*,1) stayed within the range of 0.25~0.45 with the increase of n. At the same time, the height overlap ratios of *B*(*n*,2) and *B*(*n*,3) showed increasing trends when n increased, in which that of *B*(*n*,3) were significantly larger. This is because the collapse on the negative slope side happened more noticeably as n increased, leading to insufficient support for *B*(*n*,3). The molten pool flowed down along the negative slope. Therefore, the width overlap ratio decreased and the height overlap ratio increased. The phenomenon indicates the necessity of adjusting the deposition path on the negative slope side to compensate for the severe collapse.

### 3.3. Forming Mechanism of Inclined Thick-Walled Structures

Based on the analysis from the previous section, the height overlap ratio can effectively reflect the degree of collapse in inclined structures. Therefore, the height overlap ratio changes across different layers under various processing parameters were examined. To simplify the investigation, entire layers were studied collectively rather than analyzing each bead individually. The impact of deposition speed, deposition current as well as deposition voltage on layer morphology was explored. The inclination angle was set to 55°, and other detailed processing parameters are listed in Table 3.

**Figure 8:**
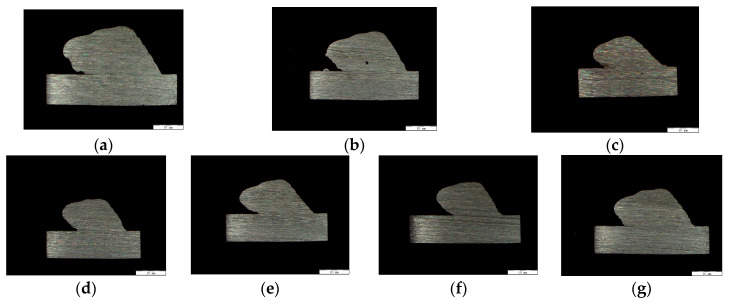
Section diagrams of the processed parts under different processing parameters: (**a**) 150 A + 4 mm/s; (**b**) 150 A + 5 mm/s; (**c**) 150 A + 6 mm/s; (**d**) 150 A + 7 mm/s; (**e**) 150 A + 8 mm/s; (**f**) 135 A + 6 mm/s, and (**g**) 165 A + 6 mm/s.

**Figure 9:**
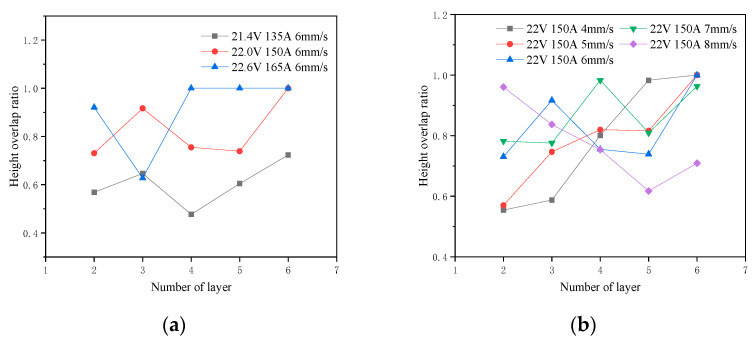
Layer height overlap ratios of inclined components under different deposition parameters: (**a**) different currents and voltages, and (**b**) different speeds.

The cross-sectional profiles of the deposited components are shown in Figure 8. The proposed approach was employed to calculate the height overlap ratio of each layer in the deposited part. The height overlap ratios for different layers under various deposition parameters are shown in Figure 9. Collapse was not prominent in the second or third layers, and height overlap occurred when the molten pool flowed onto the substrate. The height overlap ratios in the higher layers reflect the degree of collapse in the structure. The height overlap ratio increased significantly with higher deposition voltage and current but decreased as the deposition speed increased. Larger voltage and current resulted in more significant heat input, causing the molten pool to be more intensely heated, which led to more severe flow and collapse. Conversely, higher deposition speeds reduced the heating of the molten pool and provided better cooling conditions, allowing the molten pool to solidify more quickly and reducing the extent of collapse. The principles indicate that excessively large deposition currents and voltages or low deposition speeds are not suitable for depositing inclined MLMB components and if such parameters are applied, deposition path adjustments should be implemented on the earlier layers.

## 4. Discussion of Results

**Figure 10:**
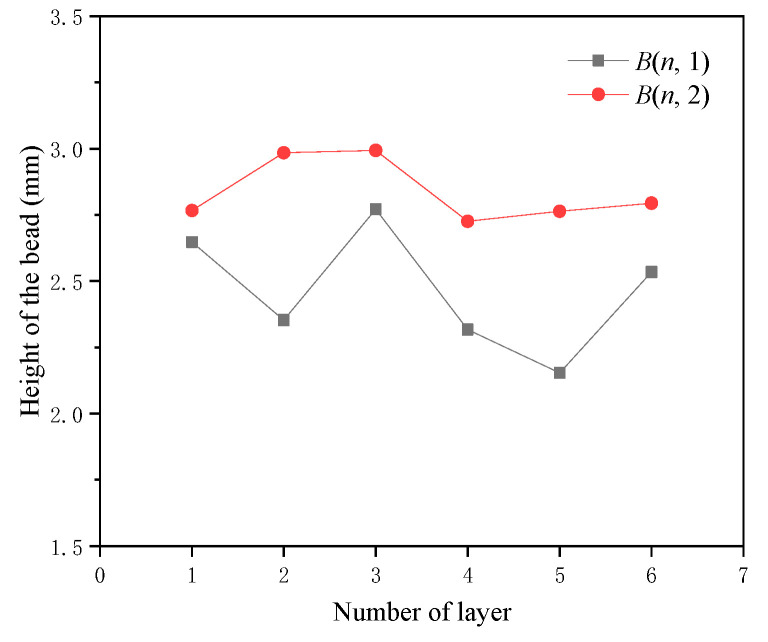
Height of *B*(*n*,1) and *B*(*n*,2) of the inclined component.

Apart from the overhanging beads, unexpected bead profiles such as widening and shortening also occurred. The heights of *B*(*n*,1) and *B*(*n*,2) are illustrated in Figure 10. The height of *B*(2,1) is lower, whereas *B*(3,1) is higher, compensating for the height deviation caused by *B*(2,1).

**Figure 11:**
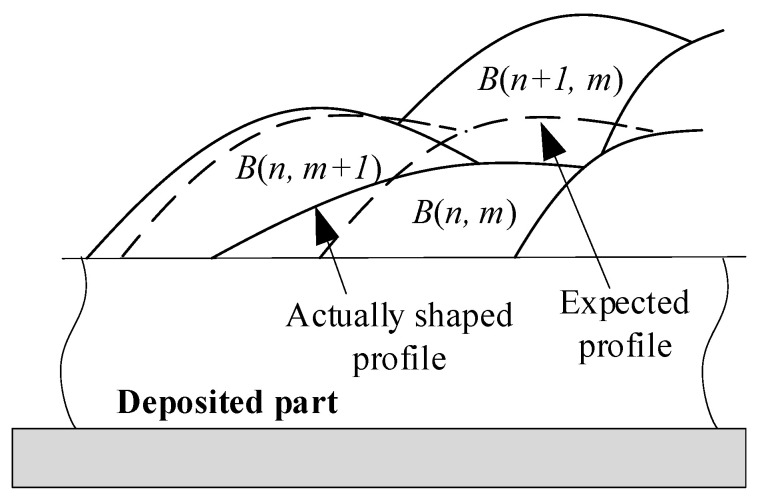
The influence of the interaction of beads on the shape formation process.

It can be observed that when a bead became wider or shorter, neighboring beads tended to exhibit an opposite change trend, compensating for the defect and preventing its accumulation. As illustrated in Figure 11, if a bead, such as *B*(*n*,*m*), exhibited widening and shortening, this shape would impact the neighboring bead *B*(*n*,*m*+1) that overlapped with *B*(*n*,*m*). The widened portion of *B*(*n*,*m*) caused *B*(*n*,*m*+1) to rise, resulting in *B*(*n*,*m*+1) being higher than its intended shape. The predetermined bead profiles of *B*(*n*,*m*) and *B*(*n*,*m*+1) are shown by the dotted lines in Figure 9, while the actual profiles are depicted by the solid lines. The increase in height of *B*(*n*,*m*+1) caused by *B*(*n*,*m*) led to a corresponding increase in the height of *B*(*n*+1,*m*), thereby partially compensating for the widening and shortening of *B*(*n*,*m*).

**Figure 12:**
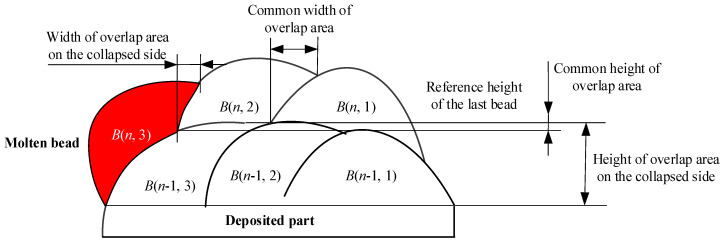
The lack of sufficiently wide base for the bead in the suspended position.

The width overlap ratios of overhanging beads were smaller than those deposited at regular positions, while the height overlap ratios were larger. This occurred because the overhanging beads lacked sufficiently wide bases for proper bead deposition, such as *B*(*n*,3) in Figure 12. Consequently, material flowed downward along the slope, resulting in decreased width overlap ratios; as the layer number increased, the width overlap ratio decreased further. A similar trend was observed in the height overlap ratios. The height overlap ratios for *B*(*n*,1) were relatively low, but due to insufficient support, *B*(*n*,3) exhibited higher height overlap ratios. As the layer number increased, the collapse on the negative slope side became more pronounced, leading to higher height overlap ratios for *B*(*n*,2) and *B*(*n*,3).

Although the heat distribution along the deposition path changed with increasing layer height, leading to deviations from the basic single deposition path model, the welding gun position remained fixed, and the processing parameters were not adjusted. The amount of material deposited per unit of time was constant, and the cross-sectional area of the deposition path remained unchanged. Consequently, all deposited material stayed within the part and the overall height of the layer did not vary significantly.

The first and last beads of a layer significantly influenced the formation of inclined MLMB structures. This impact arose from the widening and shrinking phenomena at these beads, which can lead to the deposited material adopting an unintended profile. Furthermore, as highlighted in the preceding analysis, any slight outward flow of material at the first and last beads of a given layer can result in a height deviation that becomes progressively distributed across the beads within that layer. This vertical height deviation accumulates with the addition of subsequent layers, leading to a gradual increase in the overall height deviation of the structure.

## 5. Conclusions

This paper proposed a bead profile extraction approach to explore the forming principles of wire arc additive manufacturing for the case of inclined multi-layer, multi-bead (MLMB) components. The key conclusions are summarized as follows:

- By fusing the linear-structured laser stripes captured before and after deposition, the net shape profile of each bead in an MLMB component can be extracted, which facilitates the measurement of the geometrical dimension of the deposit.
- Based on the proposed strategy, the forming mechanism of inclined thick-walled components was studied. The width overlap ratios for the beads in the suspended position of each layer were smaller than other beads while the height overlap ratios for the beads in the suspended position of each layer were larger than other beads. The height overlap ratios in the higher layers showed increasing trends with increases in deposition voltage and deposition current, as well as a decreasing trend with the increase in deposition speed.
- Except for the beads at the overhanging features, the deviation caused by a bead of unexpected profile should be compensated by the neighboring beads. The flow dynamics of the molten pool and subsequent collapse led to variations in the overlap ratios of the overhanging beads in each layer. Changes in deposition voltage, deposition current, and deposition speed affected heat distribution and cooling conditions, influencing the height overlap ratios.

## Figures and Tables

**Table 1:** Experimental setup.

Parameters	Values
Substrate material	Q235B
Substrate size	200 mm × 150 mm × 10 mm
Wire material	H08Mn2Si
Wire diameter	1.2 mm
Shielding gas composition	95%Ar + 5%CO_2_
Shielding gas flow	18 ± 0.5 L/min
Nozzle-plate distance	12 ± 0.3 mm

**Table 2:** The deposition parameters used for depositing inclined components.

Parameters	Value
Deposition voltage (V)	22
Deposition current (A)	150
Deposition speed (mm/s)	6
Single bead width (mm)	7.32
Single bead height (mm)	2.65
Distance between adjacent beads (mm)	5.4
Bead length (mm)	120
Inclination angle (°)	55
Layer offset distance (mm)	1.85

**Table 3:** The parameters used for depositing inclined components under different conditions.

NO.	Deposition Voltage (V)	Deposition Current (A)	Deposition Speed (mm/s)	Layer Offset Distance (mm)	Layer Height (mm)
1	22	150	4	1.93	2.75
2	22	150	5	1.55	2.22
3	22	150	6	1.50	2.17
4	22	150	7	1.35	1.93
5	22	150	8	1.34	1.90
6	21.4	135	6	1.35	1.92
7	22.6	165	6	1.58	2.39

## Data Availability

All data generated or analyzed during this study are included in this published article.
